# Endometrial Cell Senescence and Recurrent Spontaneous Abortion: Biomarker Potential of UCP2 and GSR

**DOI:** 10.1007/s43032-025-02023-1

**Published:** 2025-12-12

**Authors:** Zhumei Chen, Fuzhen Fang, Yan Zhang, Xiaohai Huang, Jianhuang Huang

**Affiliations:** 1 Department of Obstetrics, Affiliated Hospital of Putian University, Putian City, Fujian China; 2 Prenatal Diagnosis Center, Affiliated Hospital of Putian University, Putian City, Fujian China; 3 Department of Critical Care Medicine, Affiliated Hospital of Putian University, No.999 Dongzhen East Road, Licheng, Putian City, Fujian China; 4 Department of Neurosurgery, Affiliated Hospital of Putian University, No.999 Dongzhen East Road, Putian City, Fujian Province China

**Keywords:** Recurrent spontaneous abortion, Endometrial senescence, Oxidative stress, Immune regulation, UCP2, GSR

## Abstract

This study explores the molecular mechanisms driving recurrent spontaneous abortion, a condition affecting 1–5% of women of reproductive age, with 40–50% of cases unexplained. Focusing on endometrial cell senescence, a process linked to irreversible cell cycle arrest, the research identifies uncoupling protein 2 (UCP2) and glutathione reductase (GSR) as key contributors to this pathology. By analyzing transcriptome data from the Gene Expression Omnibus database, 21 genes associated with cellular senescence were found to be differentially expressed, with UCP2 and GSR significantly upregulated. These findings were validated using a hydrogen peroxide-induced senescence model in human endometrial stromal cells. Diagnostic potential was confirmed through receiver operating characteristic curve analysis, showing promising results for both UCP2 and GSR. The study also observed increased activity of natural killer and T cells in affected tissues, pointing to an immune component in recurrent spontaneous abortion. Drug prediction analysis highlighted dexamethasone and menadione as potential treatments. These insights suggest that oxidative stress-induced senescence plays a critical role in recurrent spontaneous abortion, with UCP2 and GSR as promising biomarkers and therapeutic targets to improve endometrial health and reduce miscarriage risk. Further clinical studies are needed to validate these findings and explore their therapeutic applications.

**Supplementary Information:**

The online version contains supplementary material available at 10.1007/s43032-025-02023-1.

## Introduction

Recurrent spontaneous abortion (RSA), defined as two or more consecutive pregnancy losses before 20 weeks, affects 1–5% of women of reproductive age [1–3]. While genetic, anatomical, hormonal, and immune factors contribute to RSA, 40–50% of cases remain unexplained, highlighting the need to explore underlying molecular pathways [4–6].

Recent studies suggest endometrial cell senescence contributes to RSA pathogenesis by upregulating markers like p16^INK4a and p21 and secreting senescence-associated secretory phenotype (SASP) factors, which disrupt the endometrial microenvironment and impair embryo implantation [7–10].The molecular pathways driving endometrial cell senescence in RSA remain poorly understood, particularly the roles of oxidative stress-regulating genes and antioxidant enzymes [6, 11, 12]. This study investigates the association of uncoupling protein 2 (UCP2) and glutathione reductase (GSR) with RSA-associated senescence using bioinformatics and in vitro models. By analyzing RNA sequencing data and validating findings in a human endometrial stromal cell (hESC) model, we aim to identify UCP2 and GSR as potential biomarkers and therapeutic targets to improve endometrial function and reduce miscarriage risk.

## Materials and Methods

### Data Sources

**Fig. 1:**
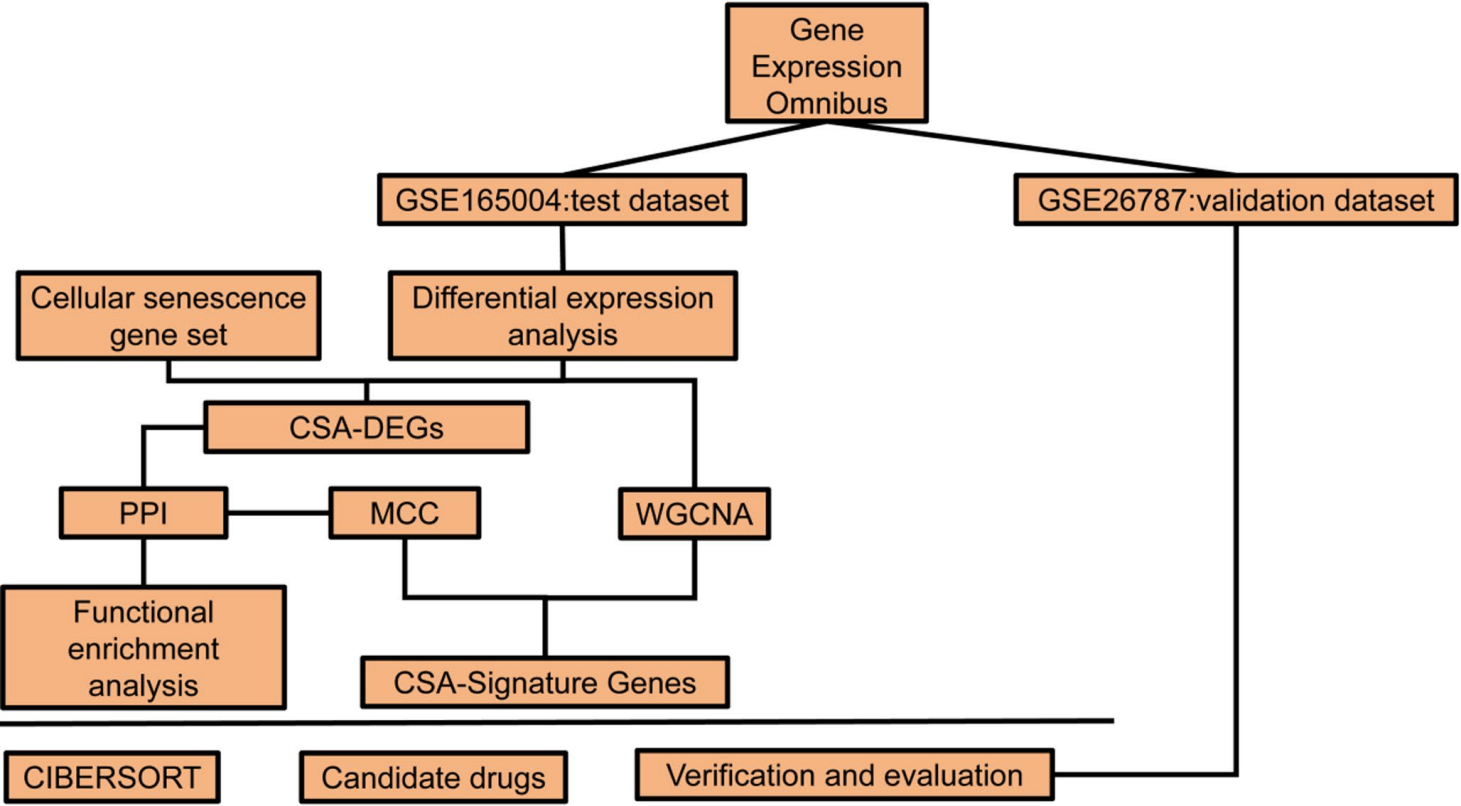
Technical roadmap of this study

We initially presented the study flowchart (Fig. 1). Transcriptome data from bulk RNA sequencing of RSA were obtained from the Gene Expression Omnibus (GEO) database. The search strategy included: (1) a search for “recurrent miscarriage”; (2) selecting Homo sapiens samples; and (3) selecting a dataset containing control samples and RSA samples. The definitions were as follows: RSA group comprised patients with two or more spontaneous miscarriages, normal parental karyotypes and miscarriages, absence of uterine abnormalities, endocrine, metabolic, autoimmune diseases or infections. Healthy controls were women with normal pregnancy terminations without a history of abortion. Two datasets, GSE165004 and GSE26787, which were based on the GPL570 platform sequencing, were chosen for this study. GSE165004 extracted endometrial sequencing data of 24 healthy control samples and 24 RSA samples, while GSE26787 extracted endometrial sequencing data of 5 healthy control samples and 5 RSA samples. The Aging Atlas database (https://ngdc.cncb.ac.cn/aging/age_related_genes) provided a list of 503 genes associated with cellular senescence.

### Analysis of Differentially Expressed Genes (DEGs)

To identify DEGs in endometrial tissues of RSA patients and normal pregnant women, we conducted differential expression analysis using the “Limma” package [13]. First, we performed principal component analysis (PCA) to identify outlier samples and normalized them using the “NormalizeBetweenArrays” function. DEGs with an adjusted *p*-value < 0.05 were considered significant. The results were visualized using box plots, PCA plots, volcano plots, and heat maps.

### Identification of Cellular Senescence-Related Genes

Cellular senescence-associated DEGs (CSA-DEGs) were obtained by visualizing the intersection of DEGs with cellular senescence-associated genes using a Venn diagram [14].

### Construction of Protein-Protein Interaction (PPI) Networks

We utilized the STRING database V12 (http://string-db.org) to construct a PPI network with CSA-DEGs. We set the “minimum required interaction score” to 0.7 and hid the unconnected nodes. The results were imported into Cytoscape software [15] V3.9.1 for visualization, and all genes were scored using the “cytoHubba” plug-in [16] to identify the top 10 genes based on the Maximum Clique Centrality (MCC) algorithm.

### Weighted Gene Co-Expression Network Analysis (WGCNA)

WGCNA is a systems biology method for identifying gene modules related to phenotypes and exploring the interrelationship between genes. We performed WGCNA using the R package “WGCNA“ [17]. First, we cleaned the GSE165004 expression data, processed missing values, and selected the top 5000 genes with the highest Median Absolute Deviation (MAD) as the data source. Next, we hierarchically clustered the samples to assess their similarity and remove outlier samples. Subsequently, we selected the optimal soft-thresholding power to construct a weighted network that satisfied the scale-free property. The parameters networkType, corType, minModuleSize, and mergeCutHeight were set to “unsigned”, “pearson”, 300, and 0.3 respectively. We employed the dynamic shear tree method to identify gene modules and calculate the correlation and significance of module feature genes with phenotypes. Finally, we identified the genes in the module most significantly correlated with the traits.

### Validation and Evaluation of CSA-Signature Genes

CSA-signature genes were obtained by intersecting the most relevant genes within the WGCNA brown module and the top 10 genes ranked by the MCC algorithm. Expression differences of CSA-signature genes were analyzed in the test set GSE165004, and their diagnostic performance was evaluated using the “pROC” package [18].

### Gene Function Enrichment Analysis

We performed GO functional enrichment and KEGG enrichment analyses of CSA-DEGs using the R package “ClusterProfiler“ [19]. The Benjamini-Hochberg method was used to correct the p-values for multiple hypothesis testing, and FDR < 0.05 was considered statistically significant.

### Analysis of Immune Cell Infiltration

Immune cell infiltration analysis was performed using the CIBERSORT algorithm [20] to estimate immune cell proportions based on gene expression data from GSE165004. The LM22 immune cell gene set served as the reference, with 1000 iterations and a significance threshold of *p* < 0.05 to ensure robust deconvolution. Spearman’s correlation analysis was applied to evaluate relationships between CSA-signature genes (UCP2 and GSR) and immune cell proportions, with a confidence cutoff of *p* < 0.05. Results were visualized using the “ggplot” package [21].

### Cell Line and Culture

The human endometrial stromal cell line (hESC, ATCC CRL-4003) was used as an in vitro model. The cell line was purchased from the China Center for Type Culture Collection in December 2024. In December 2024, short tandem repeat (STR) profiling was performed by Shanghai Biotechnology Co., Ltd., confirming genetic consistency with the hESC cell line in the ATCC database. In January 2025, the cell line was tested for mycoplasma contamination using a PCR-based mycoplasma detection kit (Takara Bio), confirming no contamination.

Cells were cultured in DMEM/F12 medium (Thermo Fisher) supplemented with 10% fetal bovine serum (FBS, Gibco) and 1% penicillin-streptomycin (Sigma-Aldrich) at 37 °C in a 5% CO₂ atmosphere. To mimic the oxidative stress environment associated with recurrent spontaneous abortion (RSA), experimental group cells were treated with 100 µM H₂O₂ (Sigma-Aldrich) for 24 h to induce senescence, while control group cells were treated with an equal volume of PBS. The H₂O₂ concentration was optimized through preliminary experiments to induce senescence without causing significant apoptosis.

### SA-β-gal Activity Assay

Cellular senescence was assessed using a commercial SA-β-gal staining kit (Cell Signaling Technology, #9860). Cells were seeded at a density of 5 × 10⁴ cells/well in 6-well plates and cultured for 48 h post-treatment. After fixation with 2% formaldehyde/0.2% glutaraldehyde, cells were incubated in X-gal solution at pH 6.0 for 16 h at 37 °C. Ten random fields were observed under a microscope (Olympus IX71), and the proportion of SA-β-gal-positive cells (blue-stained) was calculated as the number of positive cells divided by the total cell count.

### Immunofluorescence Quantitative Analysis

Cells were seeded on coverslips, treated with H₂O₂ or PBS, and fixed in 4% paraformaldehyde (Sigma-Aldrich) for 15 min, followed by permeabilization with 0.1% Triton X-100 for 5 min. After blocking with 5% BSA (Sigma-Aldrich) for 1 h, cells were incubated overnight at 4 °C with primary antibodies against p16^INK4a (1:200, Abcam, ab108349), p21 (1:200, Cell Signaling Technology, #2947), UCP2 (1:100, Santa Cruz Biotechnology, sc-390189), and GSR (1:100, Abcam, ab16801). Cells were then incubated with Alexa Fluor 488-conjugated secondary antibody (1:500, Thermo Fisher, A-11008) for 1 h, and nuclei were counterstained with DAPI (1 µg/mL, Sigma-Aldrich). Fluorescence was observed using a fluorescence microscope (Zeiss Axio Observer, fixed exposure time), with 10 random fields captured per group. Fluorescence intensity was quantified using ImageJ software (NIH, v1.53) to calculate the average fluorescence value per unit area (relative fluorescence units, RFU). Results were recorded as quantitative data.

### GSR Activity Assay

GSR enzyme activity was measured using a GSR activity assay kit (Abcam, ab83461). Total protein was extracted from cell lysates, and absorbance changes at 4–12 nm were measured according to the kit instructions to calculate GSR activity. Experiments were performed in triplicate.

### Prediction of Drug Candidates

CSA-signature genes were entered into the Enrichr website (https://www.amp.pharm.mssm.edu/Enrichr/) to obtain target drug information. Drug candidates were ranked based on the composite score, which indicates the degree of association between the small molecule drug and the gene of interest. Drugs with *p* < 0.05 and a higher composite score were considered significant.

### Statistical Analysis

Statistical analysis was conducted using R (version 4.3.2). The Wilcoxon test was used to assess expression differences between groups. SA-β-gal positive cell proportions, immunofluorescence quantitative data (RFU), and GSR activity data were expressed as mean ± standard deviation. Differences between the H₂O₂-treated and control groups were analyzed using the t-test (GraphPad Prism v9.0), with *p* < 0.05 considered statistically significant. All experiments were repeated three times.

## Results

### Cellular Senescence-Associated DEGs

**Fig. 2:**
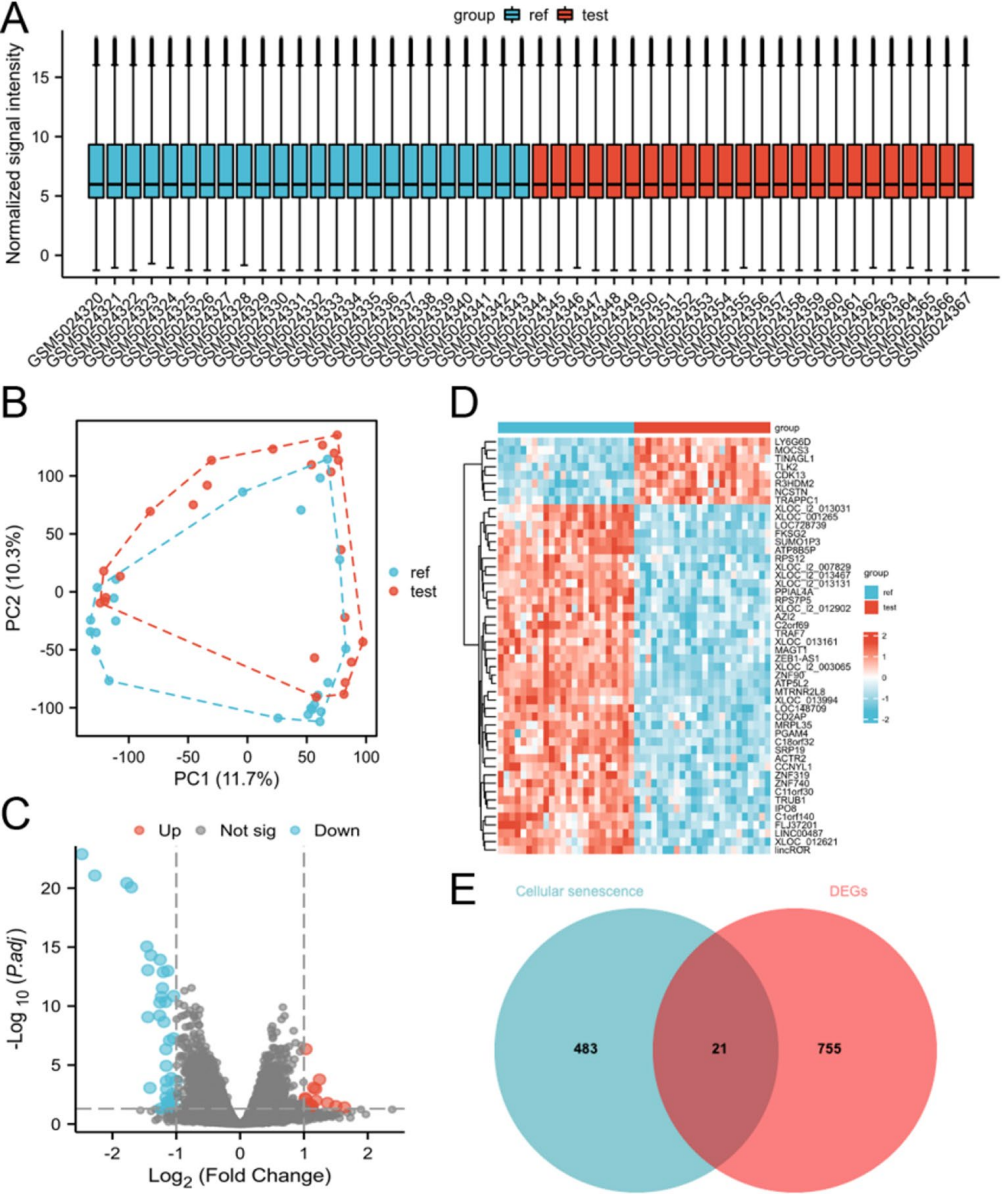
Dataset GSE165004 to obtain cellular senescence-associated DEGs. **A**-**B** All samples in dataset GSE165004 were eligible and the difference between the 2 groups was significant. **C**-**D** dataset GSE165004 obtained 776 differential genes according to the threshold. **E** Intersection of differential genes and cellular senescence-associated genes to obtain 21 cellular senescence-associated DEGs. *DEGs* differentially expressed genes

The mRNA sequencing data of control and experimental samples in dataset GSE165004 exhibited satisfactory quality, with no obvious outlier samples and significant differences between the two groups (Fig. 2A-B), thus all samples were included in the study. After differential expression analysis, we obtained 776 DEGs based on the threshold (Fig. 2C-D), of which 480 were down-regulated and 296 were up-regulated. By intersecting these DEGs with the 503 cellular senescence-associated genes, we obtained 21 CSA-DEGs (Fig. 2E).

### Construction of PPI Network and Functional Enrichment Analysis

**Fig. 3:**
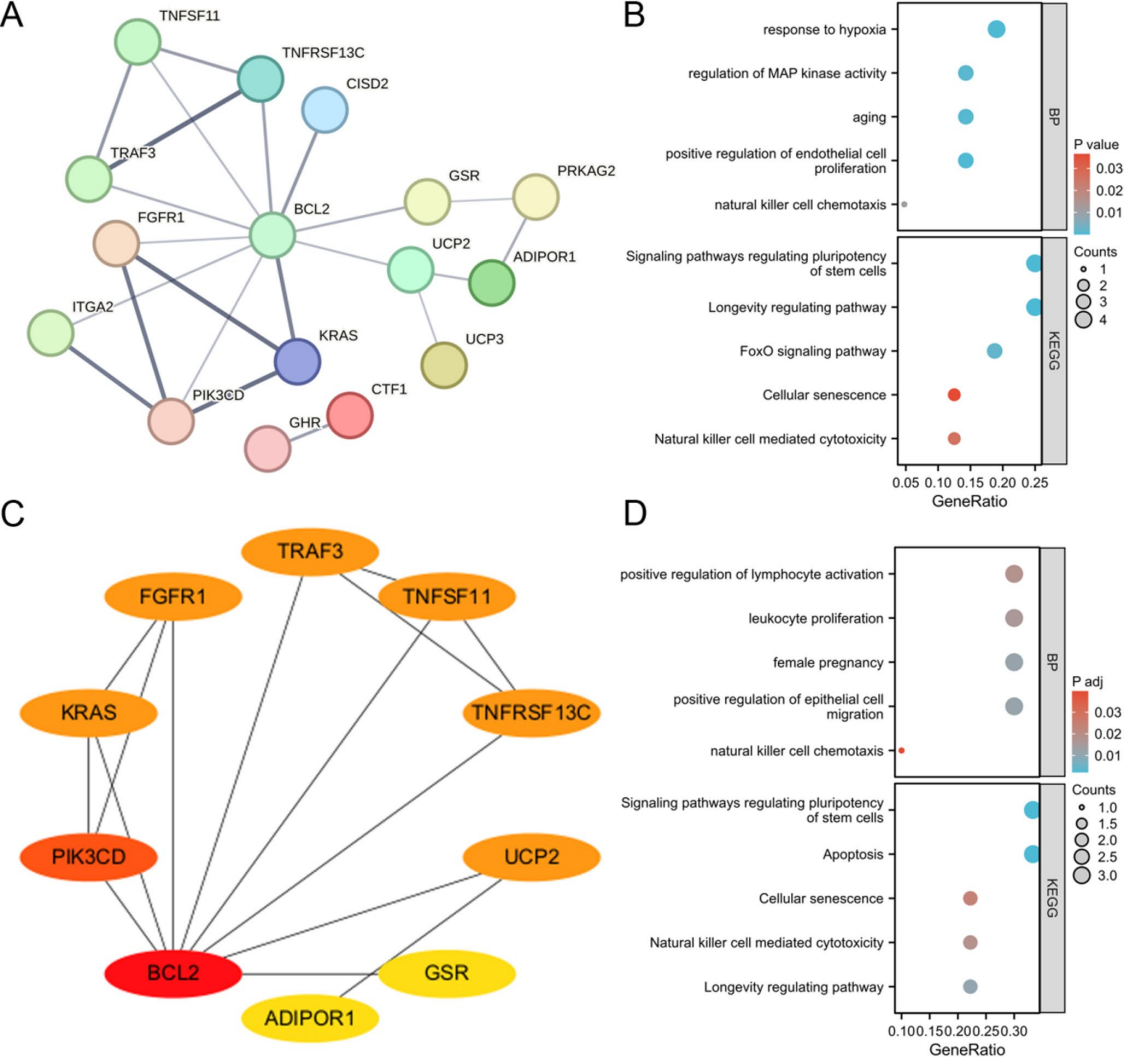
PPI network and gene function enrichment analysis based on CSA-DEGs constructed from RSA patients. **A** PPI network of CSA-DEGs constructed by STRING database. **B** Bubble plots showing the gene function enrichment results of CSA-DEGs. **C**-**D** MCC algorithm scores of the top 10 CSA-DEGs interactions network and gene function enrichment Results. *CSA-DEGs* cellular senescence-associated differentially expressed genes

We constructed the PPI network of CSA-DEGs using the STRING database, which consisted of 21 nodes and 22 edges, with a PPI enrichment p-value of 9.61e-06 (Fig. 3A). GO and KEGG enrichment analyses revealed that these genes were primarily involved in signaling pathways related to senescence, cell proliferation, and immunomodulation, particularly the Longevity regulating pathway and Cellular senescence pathway (Fig. 3B).

Using the “cytoHubba” plug-in, we scored all genes and identified the top 10 genes based on the MCC algorithm (Fig. 3C). GO and KEGG enrichment analyses of these genes showed their involvement in processes such as activation, natural killer cell chemotaxis, positive regulation of epithelial cell proliferation, and various signaling pathways including Cellular senescence, Apoptosis, and Signaling pathways regulating pluripotency of stem cells (Fig. 3D).

### WGCNA

**Fig. 4:**
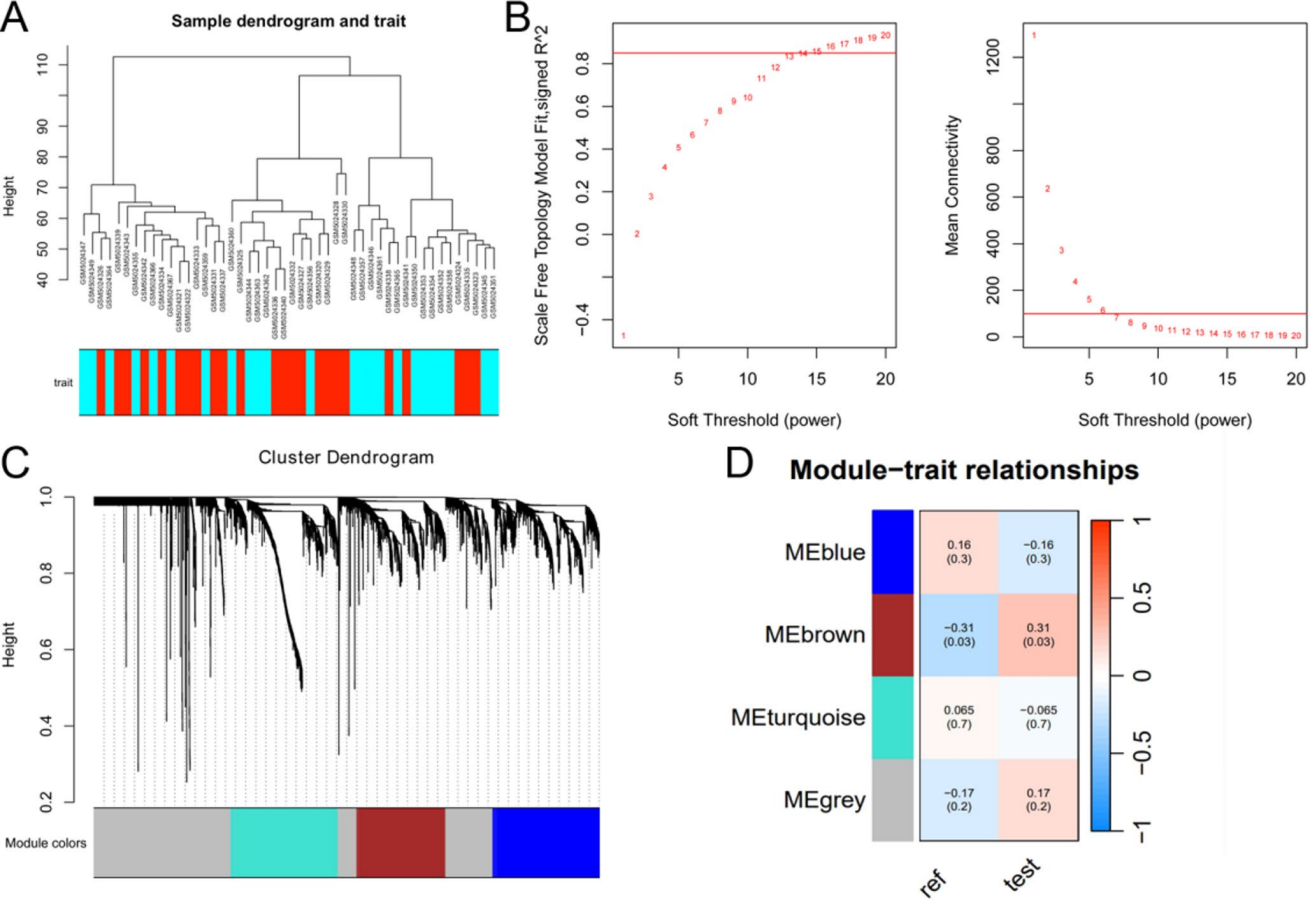
Dataset GSE165004 was subjected to WGCNA analysis to screen for the most relevant modules for the trait. **A** Pre-analysis quality control of dataset GSE165004 showed no significant outlier samples. **B**-**C** The optimal soft threshold of 14 was automatically selected based on the soft threshold map and a co-expression network was constructed using a one-step approach to identify the four modules. **D** Correlation analysis showed that the brown module had the highest correlation with the trait with the highest correlation. *WGCNA* weighted gene co-expression network analysis

In the WGCNA analysis of dataset GSE165004, we ensured the data were of good quality with no significant outlier samples (Fig. 4A). We selected an optimal soft threshold of 14 and constructed a co-expression network using a one-step method, which identified four modules (Fig. 4B-C). Correlation analysis revealed that the brown module exhibited the highest correlation with the trait (*r* = 0.31, *p* = 0.03, Fig. 4D). Subsequently, we identified 702 genes most relevant to the trait within the brown module (Supplementary Table 1).

### Identification and Validation of CSA-Signature Genes

**Fig. 5:**
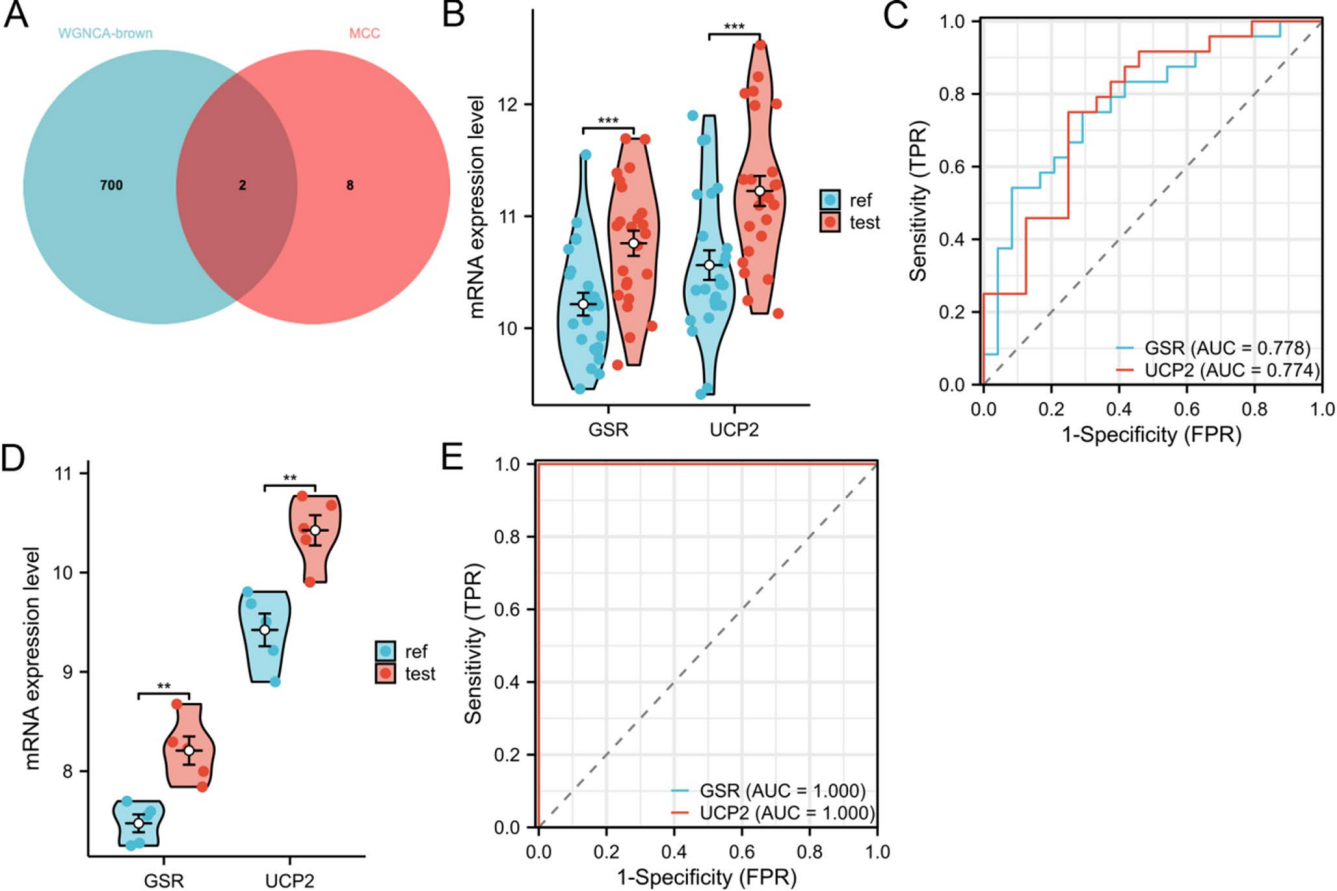
Identification and validation of CSA-signature genes. **A** Venn diagram visualizing the acquisition of CSA-signature genes. **B** In the test set dataset GSE165004, the mRNA expression ratios of *UCP2* and *GSR* in the experimental group were significantly higher than that of the control group. **C** ROC curves suggesting that *UCP2* and *GSR* have good diagnostic performance. **D**-**E** The above results were validated in the external validation dataset GSE26787, and similar results were obtained

We intersected the genes within the WGCNA brown module with the genes ranked by the MCC algorithm to obtain the CSA-signature genes of RSA, namely *UCP2* and *GSR* (Fig. 5A). Expression analysis in the test set GSE165004 demonstrated significantly higher mRNA expression ratios of *UCP2* and *GSR* in the experimental group compared to the control group (*p* < 0.001, Fig. 5B). ROC curve analysis indicated that *UCP2* (AUC = 0.774) and *GSR* (AUC = 0.778) exhibited good diagnostic efficacy (Fig. 5C). Furthermore, we validated these results in the external validation set GSE26787 (Supplementary Fig. 1 for quality control and analysis of variance), which yielded consistent findings (Fig. 5D-E).

### Immune Infiltration Analysis

**Fig. 6:**
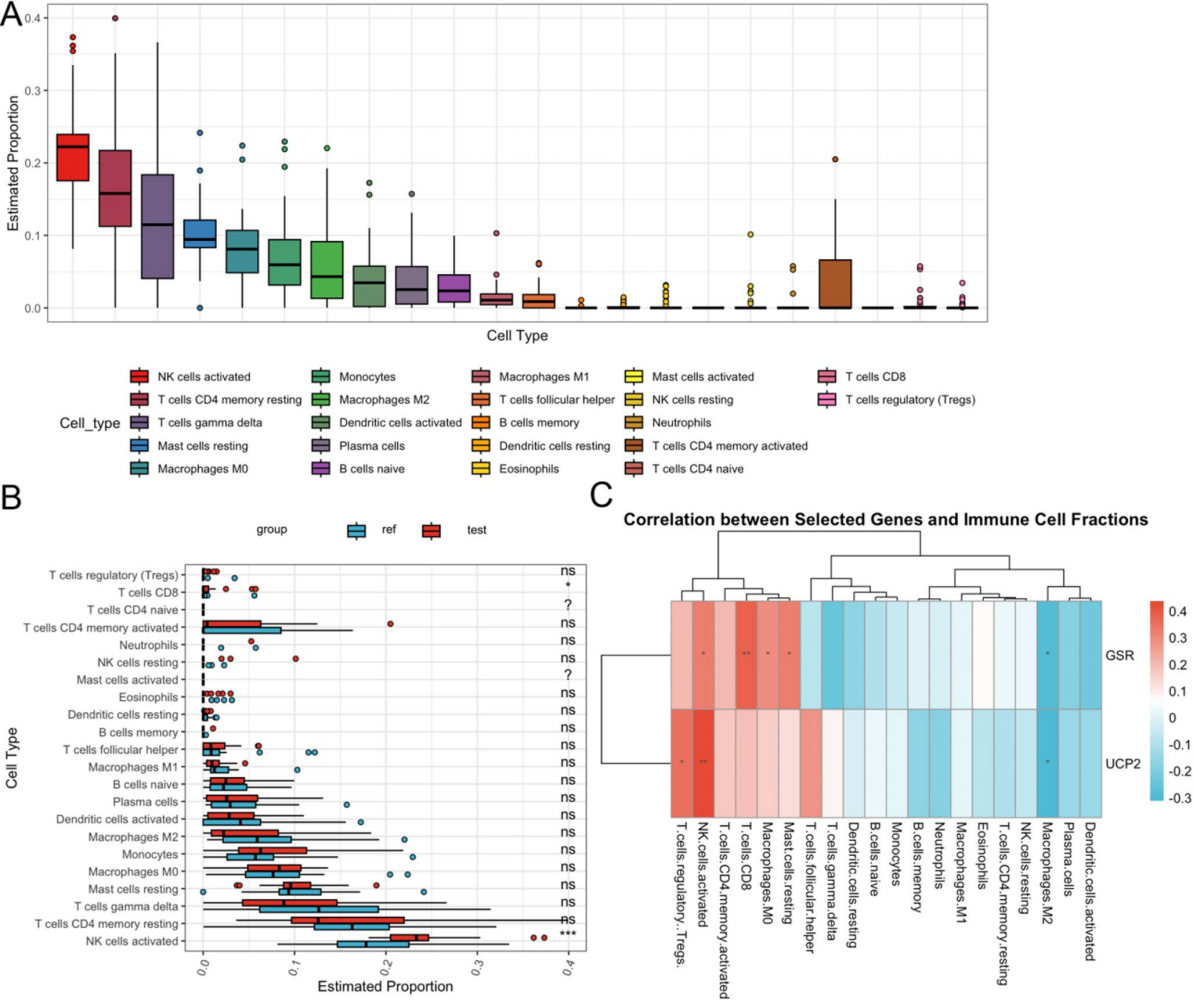
Immune infiltration analysis of dataset GSE165004. **A** Bar graph showing the abundance of each immune cell. **B** Bar graph showing the comparison of the proportion of each immune cell between the 2 groups. **C** Heatmap demonstrating the correlation of *GSR* and *UCP2* with each immune cell. *, *p* < 0.05; **, *p* < 0.01

Given the relevance of CSA-DEGs to immune function demonstrated by GO and KEGG enrichment analyses, we performed CIBERSORT analysis on the test set dataset GSE165004. The results revealed the three immune cells with the highest abundance to be activated NK cells, resting CD4 memory T cells, and gamma delta T cells (Fig. 6A). Comparison of cell proportions between the two groups indicated significantly higher proportions of activated NK cells (*p* < 0.001) and CD8 T cells (*p* < 0.05) in the experimental group compared to the control group (Fig. 6B). Furthermore, *GSR* exhibited significant positive correlations with CD8 T cells (*p* < 0.01), activated NK cells (*p* < 0.05), M0 macrophages (*p* < 0.05), and resting mast cells (*p* < 0.05), while showing significant negative correlation with M2 macrophages (*p* < 0.05). *UCP2*, on the other hand, displayed significant positive correlations with activated NK cells (*p* < 0.01) and regulatory T cells (Tregs) (*p* < 0.05), as well as significant negative correlation with M2 macrophages (*p* < 0.05) (Fig. 6C).

### Cytological Validation of Endometrial Cell Senescence

**Fig. 7:**
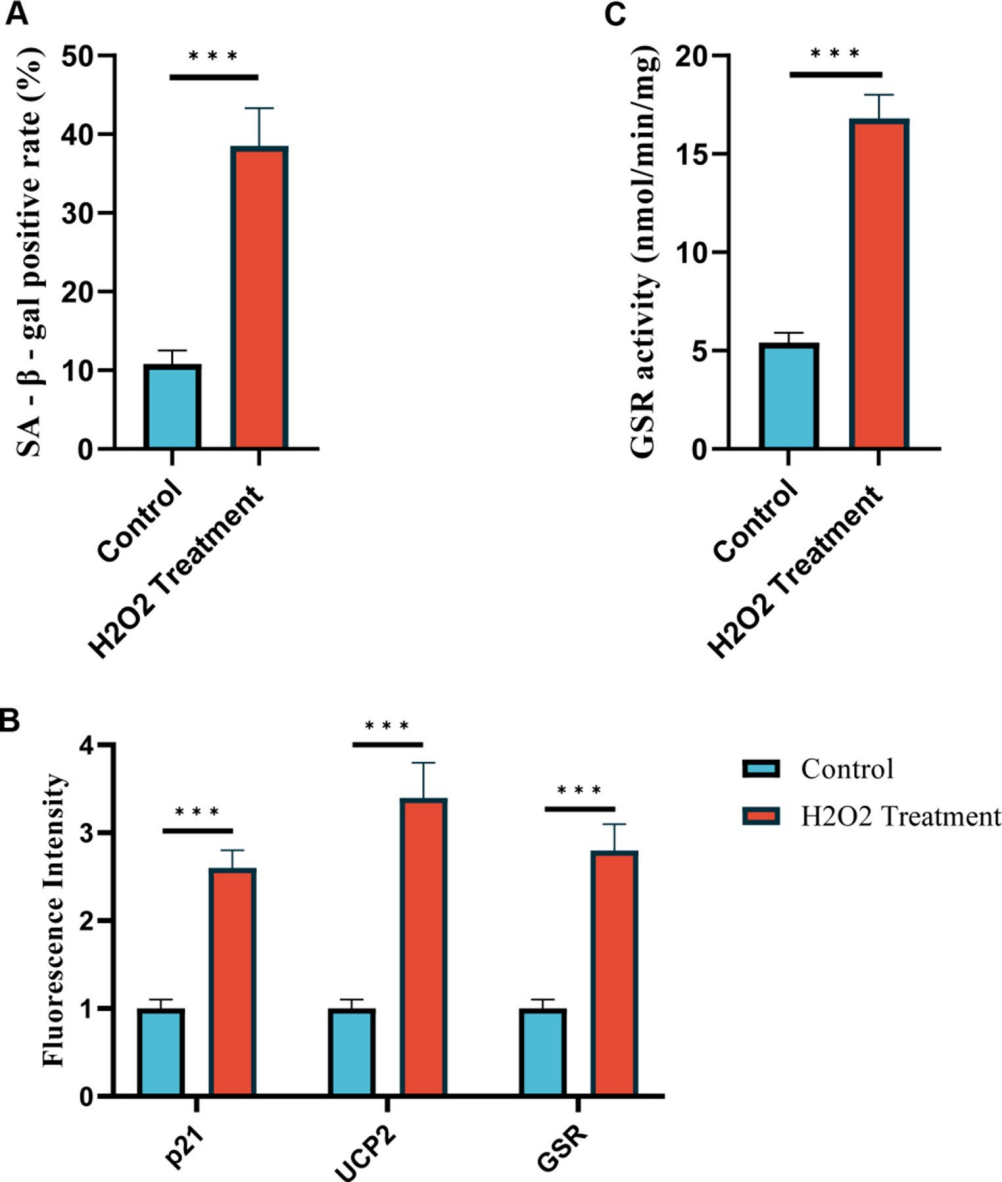
Quantitative Analysis of Senescence Markers in H₂O₂-Induced hESC Cells. **A** SA-β-gal staining revealed a higher proportion of SA-β-gal-positive cells in the H₂O₂-treated group (38.5 ± 4.8%) compared to the control group (10.8 ± 1.7%, *p* < 0.001). **B** Immunofluorescence quantitative analysis showed increased relative fluorescence intensity (RFU, normalized to control = 1.0) for p16^INK4a (2.9 ± 0.3-fold, *p* < 0.01), p21 (2.6 ± 0.2-fold, *p* < 0.01), UCP2 (3.4 ± 0.4-fold, *p* < 0.001), and GSR (2.8 ± 0.3-fold, *p* < 0.001) in the H₂O₂-treated group. **C** GSR enzyme activity assay indicated higher activity in the H₂O₂-treated group (16.8 ± 1.2 nmol/min/mg) than in the control group (5.4 ± 0.5 nmol/min/mg, *p* < 0.001)

To validate the bioinformatics findings regarding endometrial cell senescence in recurrent spontaneous abortion (RSA), we conducted in vitro experiments using an H₂O₂-induced senescence model in the hESC, without involving gene knockout or overexpression. SA-β-gal staining revealed that the proportion of SA-β-gal-positive cells in the H₂O₂-treated group (38.5 ± 4.8%) was significantly higher than in the control group (10.8 ± 1.7%, *p* < 0.001, Fig. 7A). Immunofluorescence quantitative analysis showed significantly upregulated fluorescence intensities in the H₂O₂-treated group for p16^INK4a (relative fluorescence units, RFU: 2.9 ± 0.3-fold, *p* < 0.01), p21 (2.6 ± 0.2-fold, *p* < 0.01), UCP2 (3.4 ± 0.4-fold, *p* < 0.001), and GSR (2.8 ± 0.3-fold, *p* < 0.001), with data normalized to the control group’s mean fluorescence value set as 1.0 (Fig. 7B). GSR enzyme activity assay demonstrated that GSR activity in the H₂O₂-treated group (16.8 ± 1.2 nmol/min/mg) was significantly higher than in the control group (5.4 ± 0.5 nmol/min/mg, *p* < 0.001) (Fig. 7C). These quantitative results align with the mRNA expression trends observed in the GSE165004 and GSE26787 datasets, confirming the critical roles of UCP2 and GSR in RSA-associated cellular senescence.

### Prediction of Small-Molecule Candidates

To explore potential therapeutic avenues targeting UCP2 and GSR in RSA-associated endometrial senescence, we performed small molecule prediction analysis using the DSigDB module of the Enrichr database. This analysis identified compounds significantly associated with UCP2 and GSR expression profiles (*p* < 0.05), highlighting pathways such as redox regulation and immune modulation. Dexamethasone and menadione showed high statistical significance (adjusted *p* < 0.014). Environmental compounds like cadmium and benzo[a]pyrene, also identified, underscore the role of oxidative stress in RSA pathogenesis but are not viable therapeutic candidates. These findings are hypothesis-generating, and the potential of dexamethasone and menadione as modulators of endometrial senescence requires further experimental validation. Results are summarized in Table 1.

**Table 1:** Small-molecule compounds significantly correlated with UCP2 and GSR expression profiles

Term	Adjusted *p*	Odds ratio	Combined score	Genes
dexamethasone CTD 00005779	0.014	39,158	302458.977	*GSR; UCP2*
67526-95-8 CTD 00007263	0.014	38,094	231945.332	*GSR; UCP2*
menadione CTD 00007386	0.014	37,720	216145.077	*GSR; UCP2*
CADMIUM CTD 00005555	0.014	37,716	215989.877	*GSR; UCP2*
atrazine CTD 00005450	0.032	34,064	129987.605	*GSR; UCP2*
7646-79-9 CTD 00000928	0.035	33,812	126213.939	*GSR; UCP2*
quercetin CTD 00006679	0.035	33,684	124356.610	*GSR; UCP2*
Tetradioxin CTD 00006848	0.048	32,464	108384.199	*GSR; UCP2*
Retinoic acid CTD 00006918	0.057	31,484	97413.223	*GSR; UCP2*
benzoapyrene CTD 00005488	0.061	31,152	94002.918	*GSR; UCP2*
Copper sulfate CTD 00007279	0.100	27,968	67199.787	*GSR; UCP2*
VALPROIC ACID CTD 00006977	0.176	23,376	41051.431	*GSR; UCP2*

## Discussion

This study integrates bioinformatics analysis with an H₂O₂-induced human endometrial stromal cell (hESC, CRL-4003) senescence model to explore the association between endometrial cell senescence and recurrent spontaneous abortion (RSA). We identified UCP2 and GSR as potential biomarkers for RSA-associated senescence, with their upregulation validated in both transcriptomic datasets (GSE165004, GSE26787) and in vitro experiments. These findings, supported by the widely used hESC cell line’s genetic stability and reproducibility [22–24], provide preliminary evidence for the role of oxidative stress-induced senescence in RSA pathogenesis.

### Correlation Between Cell Senescence and RSA

Differential expression analysis identified 21 cellular senescence-associated differentially expressed genes (CSA-DEGs), with a PPI network revealing their potential roles in RSA-associated senescence. GO and KEGG enrichment analyses showed enrichment in pathways related to senescence, cell proliferation, and immune regulation, including the Longevity regulating and Cellular senescence pathways. These findings align with reports that senescent cells disrupt the endometrial microenvironment, increasing miscarriage risk [25].Our in vitro experiments further validated that H₂O₂-induced hESC senescence resulted in a significant increase in SA-β-gal-positive cells (*p* < 0.001), confirming that oxidative stress effectively induces cell senescence. Immunofluorescence quantification demonstrated a significant upregulation of p16^INK4a, p21, UCP2, and GSR in the H₂O₂ treatment group (*p* < 0.001). Additionally, GSR enzyme activity was significantly enhanced in the H₂O₂ group compared to controls (*p* < 0.001). These results corroborate the mRNA expression trends observed in the GSE165004 and GSE26787 datasets, confirming the critical role of cell senescence in RSA and suggesting that UCP2 and GSR may be core regulatory factors. Furthermore, studies on RSA have identified differentially expressed genes related to immune regulation, which may play crucial roles in modulating maternal immune responses [26].

### Role of UCP2 and GSR in RSA

We validated the upregulation of UCP2 and GSR in an H₂O₂-induced hESC senescence model, consistent with transcriptomic data from GSE165004 and GSE26787 (*p* < 0.001). ROC curve analysis showed good diagnostic potential for UCP2 (AUC = 0.774) and GSR (AUC = 0.778) in distinguishing RSA from controls. These findings, supported by literature on UCP2 and GSR in oxidative stress and senescence [27–31], suggest their potential as biomarkers for RSA. However, their causal roles remain to be confirmed through functional studies, such as gene knockdown or overexpression, which were not feasible due to resource constraints.

Moreover, significant changes in immune cell infiltration patterns, particularly enhanced activation of NK and T cells, were observed in the endometrium of RSA patients. These changes in immune infiltration may be closely related to the upregulation of GSR and UCP2, influencing the pathological mechanisms of RSA. Specifically, the upregulation of UCP2 and GSR may induce local inflammation by regulating the SASP, promoting NK cell activation [32], which leads to immune imbalance in the endometrium and embryo implantation failure [33]. Enhanced T cell activation may further exacerbate inflammatory responses, impacting pregnancy immune tolerance [34]. Therefore, the upregulation of UCP2 and GSR may not only serve as markers of oxidative stress response but also drive the pathological processes of RSA through SASP and immune regulation [35, 36].

Based on this, we hypothesize that upregulation of GSR and UCP2 is a key feature of endometrial cell senescence, closely linked to the pathogenesis of RSA. During embryo implantation and development, systemic inflammatory responses increase oxidative stress, elevating ROS and RNS levels [37]. The increased GSR expression in the endometrium reflects an adaptive response to this high oxidative environment. However, sustained oxidative stress may lead to cellular dysfunction and accelerated aging [38]. Concurrently, UCP2 upregulation may promote senescence by reducing mitochondrial membrane efficiency and disrupting energy metabolism [31]. Although this compensatory response may offer short-term protection, prolonged effects can cause metabolic imbalance and energy depletion. Moreover, GSR and UCP2 upregulation may regulate the senescence-associated secretory phenotype (SASP), triggering local inflammation and tissue remodeling that impair endometrial integrity and embryo implantation capacity [39, 40]. Additionally, enhanced activation of NK and T cells may further reduce uterine receptivity and adversely affect pregnancy outcomes [32, 36]. Therefore, given the critical roles of GSR and UCP2 in RSA, they present potential therapeutic targets; modulating their expression could improve endometrial function and increase implantation success.

### Potential Application of Candidate Drugs

Drug prediction analysis identified small molecules associated with UCP2 and GSR, suggesting potential modulation of redox and immune pathways in RSA. Dexamethasone, a glucocorticoid, may reduce inflammation and improve endometrial receptivity [41, 42], while menadione, a redox modulator, could influence senescence-related oxidative stress [43]. These findings are hypothesis-generating, as environmental compounds like cadmium highlight oxidative stress but are not therapeutic candidates. Clinical validation of dexamethasone and menadione is essential to confirm their therapeutic potential.

### Strengths and Limitations of the Study

This study integrates bioinformatics and in vitro experiments to systematically validate the roles of UCP2 and GSR in RSA-related cell senescence and offers potential therapeutic targets through drug prediction. The H₂O₂-induced hESC model successfully replicated the senescence phenotype associated with RSA, and the experimental data align closely with public datasets. We acknowledge that the reliance on datasets with limited sample sizes (GSE165004 and GSE26787) may affect the robustness of the analyses; however, rigorous quality control and consistent validation of UCP2/GSR trends enhance the reliability of our findings. Future studies with larger clinical cohorts are needed to further verify the immune infiltration patterns (analyzed by CIBERSORT) and generalizability of the results.

A key limitation of this study is the use of the immortalized hESC cell line (CRL-4003), which may differ from primary endometrial cells in senescence responses and SASP profiles. Primary cells better reflect in vivo conditions but are challenging to obtain due to ethical and logistical constraints. The hESC cell line, validated by STR profiling and mycoplasma testing, has been widely used to study endometrial biology and oxidative stress [22–24]. Its standardized nature ensured reproducible results, with senescence markers (SA-β-gal, p16^INK4a, p21, UCP2, GSR) aligning with transcriptomic trends, supporting its suitability for this exploratory study. Future validation in primary cells or patient-derived tissues is needed to enhance translational relevance.

## Conclusion

This study, through the integration of bioinformatics analysis and an H₂O₂-induced hESC cell senescence model, elucidates the molecular mechanisms of endometrial cell senescence in RSA. The significant upregulation of SA-β-gal, p16^INK4a, p21, UCP2, and GSR, along with enhanced GSR enzyme activity, confirms the critical role of oxidative stress-induced cell senescence in RSA. As CSA-signature genes, UCP2 and GSR are significantly upregulated both in protein expression and function, and they may drive endometrial microenvironment imbalance through the regulation of mitochondrial function, oxidative stress, and SASP, thereby impacting embryo implantation. Drug prediction analysis further suggests candidates such as dexamethasone and menadione as potential therapeutic strategies targeting UCP2 and GSR. This study provides new insights into the pathological mechanisms of RSA and lays the groundwork for developing targeted therapies aimed at improving endometrial receptivity and reducing RSA incidence. Future research should further validate the regulatory mechanisms of UCP2 and GSR and their therapeutic potential in the clinical management of RSA.

## Supplementary Information

Below is the link to the electronic supplementary material.

Supplementary Material 1

Supplementary Material 2

## Data Availability

The datasets generated and analysed during the current study are available in the NCBI Gene Expression Omnibus (GEO) repository. 1. GSE165004: https://www.ncbi.nlm.nih.gov/geo/query/acc.cgi?acc=GSE165004. This dataset comprises bulk RNA sequencing data from endometrial samples. 2. GSE26787: https://www.ncbi.nlm.nih.gov/geo/query/acc.cgi?acc=GSE26787. This dataset also features bulk RNA sequencing data from endometrial samples. 3. Aging Atlas Database: https://ngdc.cncb.ac.cn/aging/age_related_genes. The cytology experiment data can be obtained from the corresponding author upon request.

## Abbreviations

UCP2: Uncoupling Protein 2
GSR: Glutathione Reductase
DEGs: Differentially Expressed Genes
CSA-D: Gs Cellular senescence-associated differentially expressed genes
WGCNA: Weighted Gene Co-expression Network Analysis
PPI: Protein-protein Interaction
GO_BP: Gene Ontology Biological Process
KEGG: Kyoto Encyclopedia of Genes and Genomes
RSA: Recurrent miscarriage
