# Supplementary material for: Endometrial Cell Senescence and Recurrent Spontaneous Abortion: Biomarker Potential of UCP2 and GSR
Source: Reprod Sci. 2025 Dec 12;33(1):161–73. doi: 10.1007/s43032-025-02023-1 (PMC12948855; doi:10.1007/s43032-025-02023-1)
Supplement: Supplementary file 2 — Supplementary Material 2 [file 43032_2025_2023_MOESM2_ESM.docx]

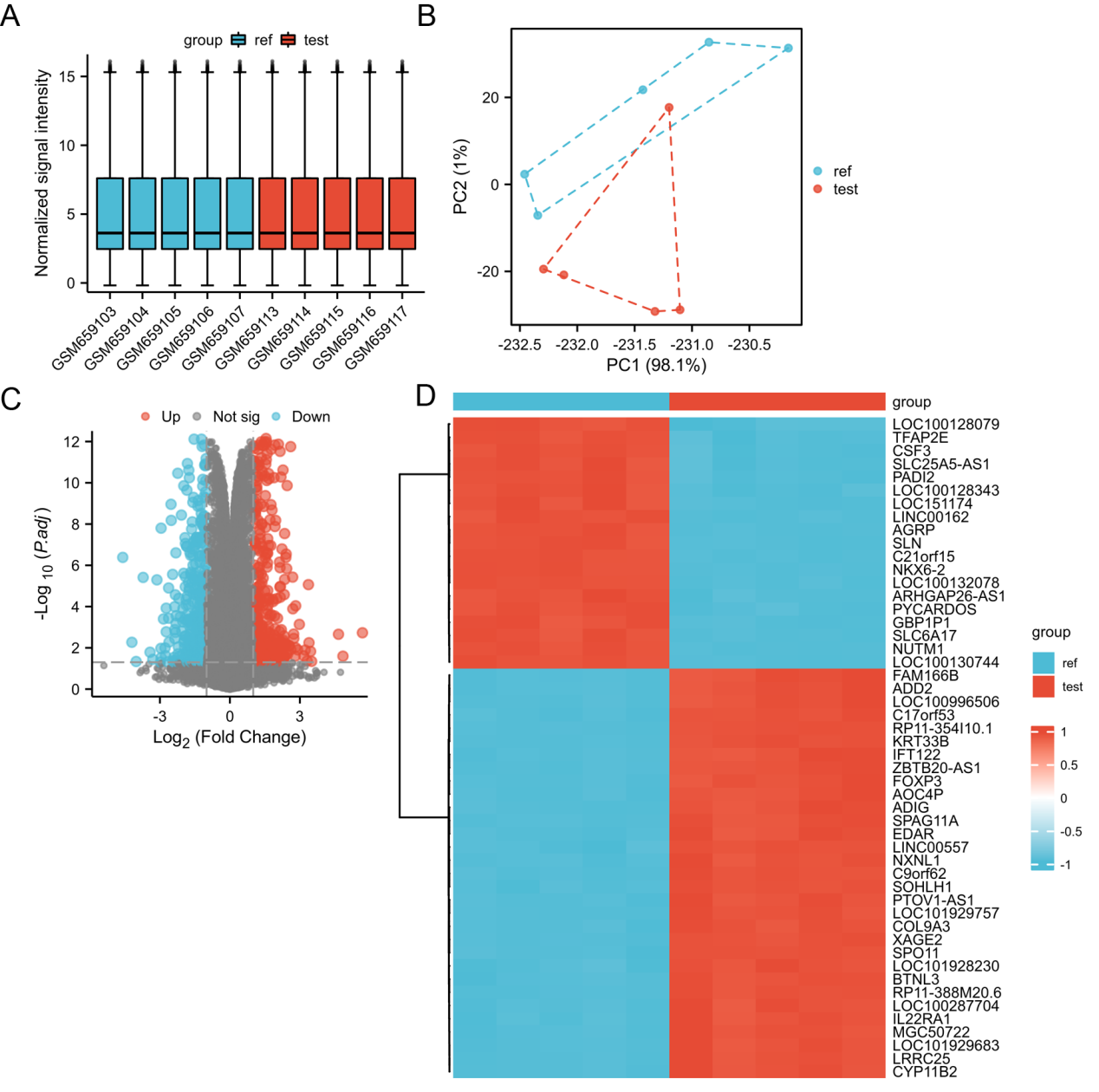


**Supplementary Figure 1. Quality Control and DEGs Analysis of Dataset GSE26787.**A-B. All samples in dataset GSE26787 were eligible and the difference between the 2 groups was significant. C-D.The volcano plot and heatmap illustrated the results of the differential expression analysis for dataset GSE26787. DEGs : Differentially Expressed Genes.
